# Additive effects of cherlerythrine chloride combination with erlotinib in human non-small cell lung cancer cells

**DOI:** 10.1371/journal.pone.0175466

**Published:** 2017-04-11

**Authors:** Miao He, Zhaoying Yang, Le Zhang, Changlong Song, Youjun Li, Xingyi Zhang

**Affiliations:** 1 Department of anesthesia, The Second Hospital of Jilin University, Changchun, China; 2 Department of breast surgery, China-Japan Union Hospital of Jilin University, Changchun, China; 3 Department of anatomy, Norman Bethune Health Science Center of Jilin University, Changchun, China; 4 Department of thorax surgery, The Second Hospital of Jilin University, Changchun, China; Universita degli Studi di Parma, ITALY

## Abstract

Several studies implicate that lung cancer progression is governed by the interaction between epidermal growth factor receptor (EGFR) signaling and protein kinase C (PKC) pathways. Combined the targeting of EGFR and PKC may have an additive or synergistic effects in lung cancer treatment. The aim of this study is to explore the potential utility by inhibiting these two pathways with the combination of erlotinib and chelerythrine chloride in non-small cell lung cancer (NSCLC) cell lines. The erlotinib-less sensitive cell lines SK-MES-1 and A549 were treated with erlotinib or chelerythrine by themselves or in combination with each other. The cell viability, clonogenic survival, cell migration, invasion, cell apoptosis effects and immunoblotting were accessed *in vitro*. Tumor growth was evaluated *in vivo*. There were additive effects of chelerythrine combined with erlotinib treatment in all NSCLC cell lines, resulting in a significant decrease in cell viability, clonogenicity, migratory and invasive capabilities as well as in the induction of apoptosis. Concordantly, the combined treatment caused a significant delay in tumor growth. The treatment effectively blocked EGFR signaling through decreasing phosphorylation of downstream targets such as STAT3, ERK1/2, p38 MAPK and Bad proteins. Our study supports the functional interaction between the EGFR and PKC pathways in lung cancer and provides a clinically exploitable strategy for erlotinib-less sensitive non-small cell lung cancer patients.

## Introduction

Lung cancer is the leading cause of cancer deaths worldwide [1], and the 5-year survival rate for all stages is only 15% [2]. In China, lung cancer is the leading cancer among all cancers, followed by cancers of the stomach, liver, colorectum, and breasts [3]. There are two major forms of lung cancer: non-small-cell lung cancer (NSCLC), which accounts for 85% of all lung cancers, and small-cell lung cancer, which accounts for the remaining 15%. Based on cell morphology, NSCLC can be subdivided into three categories: squamous-cell carcinoma (SQCLC), adenocarcinoma, and large-cell lung cancer. Regardless of the efforts made in advanced detection and treatments, NSCLC is often diagnosed at advanced stages and has a poor prognosis [4]. Unfortunately, standard treatment modalities such as chemotherapy, radiotherapy, and surgery have reached a plateau [5]. Therefore, identifying new effective therapeutic treatments for lung cancer is critical.

Targeting the epidermal growth factor receptor (EGFR) has played a central role in advancing NSCLC research and treatment over the last several years. Erlotinib is an EGFR tyrosine kinase inhibitor (TKI), which is widely used in clinical studies, and its therapeutic effect has been confirmed in a specific cohort of lung cancer subjects. However, it is expensive and its efficacy is limited by primary or secondary drug resistance which develops over extended periods of treatments. The mechanisms behind the resistance to TKIs are only partially understood. Mahlet et al. found that the erlotinib-resistant cell line has a remarkable PKCα up-regulation and PKCδ down-regulation signaling [6].

Recent evidence suggests the PKC pathway is involved in lung cancer. It has been well known that the Protein Kinase C (PKC) can regulate the PI3K/AKT pathway both positively [7] and negatively [8], depending on the isoform. Certain PKC isoforms are exclusively expressed and activated in NSCLC cells. Moreover, Rottlerin, a PKCδ inhibitor, has shown to increase apoptosis and sensitize NSCLC cells to other chemotherapeutic agents [9]. Additionally, the overexpression of some PKC family members has also been associated with low sensitivity to the irreversible TKI afatinib in lung cell line models [10]. Thus, PKC inhibitors are one of the promising agents against NSCLC.

Chelerythrine chloride is a benzophenanthridine alkaloid extracted from the bark of the Southern Prickly Ash, *Zanthoxylum clava-herculis*. Although having numerous documented biological effects, it is known for its specific inhibiting activities against PKC [11]. It has been reported that chelerythrine chloride is cytotoxic to a variety of cancer cell lines: MCF-7 and MCF-7ADR (breast), DaOY (brain), LnCaP (prostate), SQ-20B, JSQ-3, SCC-35 and SCC61 (head neck squamous cell carcinoma) [12].

Because it targets a particular receptor it may not be sufficient for long-term control of lung cancer due to compensatory feedback loops. Therefore, we hypothesize that the abnormal or unscheduled activation of the development of the important PKC signaling pathway may be associated with NSCLC progression and the combination of the EGFR inhibitor erlotinib with the PKC inhibitor chelerythrine may be a potential therapeutic strategy in NSCLC.

## Materials and methods

### Reagents

Chelerythrine chloride was purchased from Santa Cruz (Dallas, TX, USA), and erlotinib was purchased from the LC Lab (Woburn, MA, USA). All compounds were reconstituted in dimethyl sulfoxide (DMSO) (Sigma Aldrich, St. Louis, MO, USA) and filter sterilized. All antibodies for western blotting were purchased from Cell Signaling (Danvers, MA, USA).

### Cell lines

SK-MES-1 cells, a human lung squamous carcinoma cell line; A549 cells, a human adenocarcinoma cell line and HCC827, a lung adenocarcinoma with an acquired mutation in EGFR tyrosine kinase domain (E746-A750 deletion) were purchased from American Type Culture Collection (Manassas, VA, USA). SK-MES-1 cells were cultured in an Eagle’s modified medium (Gibco, Rockville, MD, USA), and the A549 cells were cultured in a DMEM H21 medium (Gibco). The growth medium contained 10% heat-inactivated fetal bovine serum (Gibco), 0.03% L-glutamine, 100 units/mL penicillin, and 3mg/mL of streptomycin at 37°C in a humidified atmosphere with 5% CO_2_. The HCC827 cells were cultured in a RPMI 1640 supplemented with a 10% fetal bovine serum (FBS).

### IC_50_ and cell viability

Cells in mid-log phase were used in all experiments. To determine the IC_50_ for erlotinib or chelerythrine, SK-MES-1 cells and A549 cells were plated into 96-well microplates (4×l0^3^ cells/well) in a serum-containing media overnight. Erlotinib (0, 2.5, 5, 10, 25 or 50μM), chelerythrine (0, 1.25, 2.5, 5, 7.5 or 10μM) were added to the culture medium. Equal concentrations of solvents (DMSO) were distributed among all the wells and cultured for 48 hours. Cell viability was assessed by incubation with 10% alamar blue (Invitrogen, Carlsbad, CA, USA) for 4 hours. Photo absorbance (A) was measured at wavelengths of 540 to 590nm, for each plate with an Optimax Microplate reader (Molecular Devices, Sunnyvale, CA, USA). Similarly, the IC_50_ for erlotinib or chelerythrine on HCC827 cells was also determined the same as the methods described above.

After IC_50_ was chosen, the effect of the combination of erlotinib and chelerythrine on SK-MES-1 and A549 cells was performed as previously described [13]. The cells (4×l0^3^ cells/well) were plated into 96-well microplates overnight. Erlotinib 5μM with chelerythrine (2.5, 5, 7.5 and 10μM) or chelerythrine 2.5μM with erlotinib (2.5, 5, 10 or 20μM) were added to the culture medium. Photo absorbance (A) was measured daily by alamar blue assay for 24, 48, and 72 hours. The percentage of cell viability was calculated as following: cell viability (%) = (A_drug_ − A_blank_) /(A_control_ − A_blank_) ×100%.

### Combination effects

Combination effects for co-exposure on potency were evaluated using two methods: 1) The Bliss independence criterion and 2) The combination index (CI). The main assumption of the Bliss independence criterion is that two or more toxic agents act independently from one another. Drug 1 at dose x produces a response E(x), and drug 2 at dose y produces a response E(y). Next, the two drugs are combined at the dose pair(x, y) and combination response E(x,y) is observed. If the combined effect is higher than the expected value from E(x,y), the interaction is synergistic, while if this effect is lower, the interaction is antagonistic, and if the value is equal, the interaction is additive [14]. In general, a CI value of less than 0.9, between 0.9 and 1.1, or greater than 1.1 indicates synergy, additivity, or antagonism, respectively. IC_50_(a) is the IC_50_ of inhibitor A; IC_50_(b) is the IC_50_ for inhibitor B; Da is the concentration of inhibitor A in combination with inhibitor B that inhibited 50% of cell growth; and Db is the concentration of inhibitor B in combination with inhibitor A that inhibited 50% of cell growth [15].

E(x,y)=E(x)+E(y)−E(x)E(y)

$$CI=\;\frac{Da}{IC50\left(a\right)}+\frac{Db}{IC50\left(b\right)}+\frac{Da\times Db}{IC50\left(a\right)\times IC50\left(b\right)}$$

### Cell number counter

The same number (4×10^4^) of SK-MES-1 and A549 cells were cultured for 24 hours. The medium was replaced with fresh medium containing erlotinib, chelerythrine or combination of both. Direct cell number counting was performed after 24, 48, and 72 hours. Three independent experiments were performed.

### Cell colony formation assay

Cells in the mid-log phase were digested into a single cell suspension and inoculated into 6-well plates. Each well was inoculated with 400 cells. After 24 hours, when the cells adhered to the plate, the medium was removed and chelerythrine and/or erlotinib were applied to treat the cells for 24 hours. Subsequently, the medium was changed to a fresh growth medium and the cells were incubated for 14 days. Then, the cells were fixed with methanol and stained with Giemsa. The colonies including >50 cells were calculated. The experiments were repeated independently three times.

### Migration and invasion assay

Cell migration and invasion were assessed using BD BioCoat Matrigel Invasion Chambers and Control Inserts (BD Biosciences, San Jose, CA, USA). The same number cells were plated in 6cm plates with a growth medium cultured for 24 hours. The medium was removed and erlotinib and/or chelerythrine were applied to treat the cells for 24 hours. The viable cells were collected and seeded on either control inserts (a polyethylene terephthalate membrane) or trans-well chambers. A 2ml medium supplemented with 15% FBS was added to the lower chamber, which served as the chemoattractant. 4×10^4^ cells were re-suspended in the media plus 1% FBS was added to the upper chamber (0.5ml). 20 hours later, migrating or invading cells attached to the lower surface of the membrane insert which were fixed and stained and then counted under a microscope [16]. Relative migration was calculated by comparing cells transfected with the negative control. The percentage invasion was calculated based on the number of cells which had invaded through the Matrigel insert, divided by the number of cells which had migrated through the control insert.

### Flow cytometry apoptosis

Four experimental groups were included in the analysis: control, erlotinib, chelerythrine, and erlotinib combined with chelerythrine which were added to the culture medium. After being treated for 24 hours, cells were harvested using 0.25% trypsin. The cells were washed twice with pre-chilled PBS (4°C), and re-suspended in a 500μl binding buffer to a final cell concentration of 1-5x10^5^cells/ml. They were incubated in the dark with a 5μl Annexin-V and 5μl propidium iodide (Biolegend, San Diego, CA, USA) solution of 20μg/ml for 30 minutes. Subsequently, the suspension was analyzed using a FACScalibur (Becton Dickinson).

### Western blot analysis

Cells were harvested in a lysis buffer (0.5% sodium deoxycholate, 0.1% SDS, 1% Nonidet P-40, and 1× PBS) containing proteinase inhibitors (100μg/ml phenylmethylsulfonylfluoride, and 12μg/ml aprotinin) and 1mM of sodium orthovanadate (Sigma-Aldrich) [17]. Cell lysates were prepared and quantified for protein concentration by the Bio-Rad (Hercules, CA) method. Proteins (25mg/well) were separated with 10% or 12% SDS-PAGE gel and then transferred to a nitrocellulose membrane. After blocking with 5% BSA in 0.1% Tween 20/TBS, membranes were incubated with one of the following primary antibodies (Cell Signaling, Danvers, MA, USA) at 4°C for overnight: pSTAT3(S727)/STAT3, p-p38 MAPK/p38 MAPK, p-ERK1/2/ERK1/2, p-Bad(S112)/Bad, PARP and EGFR. The protein β-tubulin and GAPDH were used as a loading control. As secondary antibodies, either anti-mouse or anti-rabbit antibodies were used conjugated with horseradish peroxidase (Santa Cruz, Dallas, TX, USA). Immunoreactivity was detected by enhanced chemiluminescence (Denville Scientific, South Plainfield, NJ, USA)) and visualized by autoradiography.

### Ethics statement

This study was approved by the Animal Care and Use Committee of the China-Japan Union Hospital of Jilin University (No. JU20140623). All animals used in this study were in accordance with the approved guidelines of the Animal Care and Use Committee of the China-Japan Union Hospital of Jilin University.

### Xenotransplantation and drug treatment

Twenty-four male NOD SCID mice with an average body weight of 18-20g (aged 4–6 weeks) were housed in a specific pathogen free animal facility. All manipulations (i.e. handling, invasive procedures and tumor volume measurements) were performed in a laminar flow hood under strict sterile conditions. Mice were injected with 4×10^6^ cells (SK-MES-1) suspended in 0.1ml PBS subcutaneously into the left chest. Mice were monitored daily until detection of the first tumor nodule. Treatment with erlotinib or/and chelerythrine was initiated when the tumor volume reached 150mm^3^ in size. Twenty-four mice were randomly assigned to one of four experimental groups (n = 6 per group): control; erlotinib (50mg/kg, o. p. daily); cheleryhine (10mg/kg, o. p. daily); and erlotinib combined with chelerythrine (50mg/kg & 10mg/kg, o. p. daily). At the end of the 14 day treatment, the mice were euthanized by CO2 asphyxiation, and the tumors and organs were removed.

### Statistical analysis

Data are represented as a means ± SD. Statistical analysis was carried out by ANOVA followed by the Dunnett's *t* test/Bonferroni multiple comparison test, considering P<0.05 to denote significant differences. The statistical analysis of *in vivo* study was carried out by Two-way ANOVA in GraphPad Prism.

## Results

### Chelerythrine potentiated antitumor effects of erlotinib

**Fig 1:**
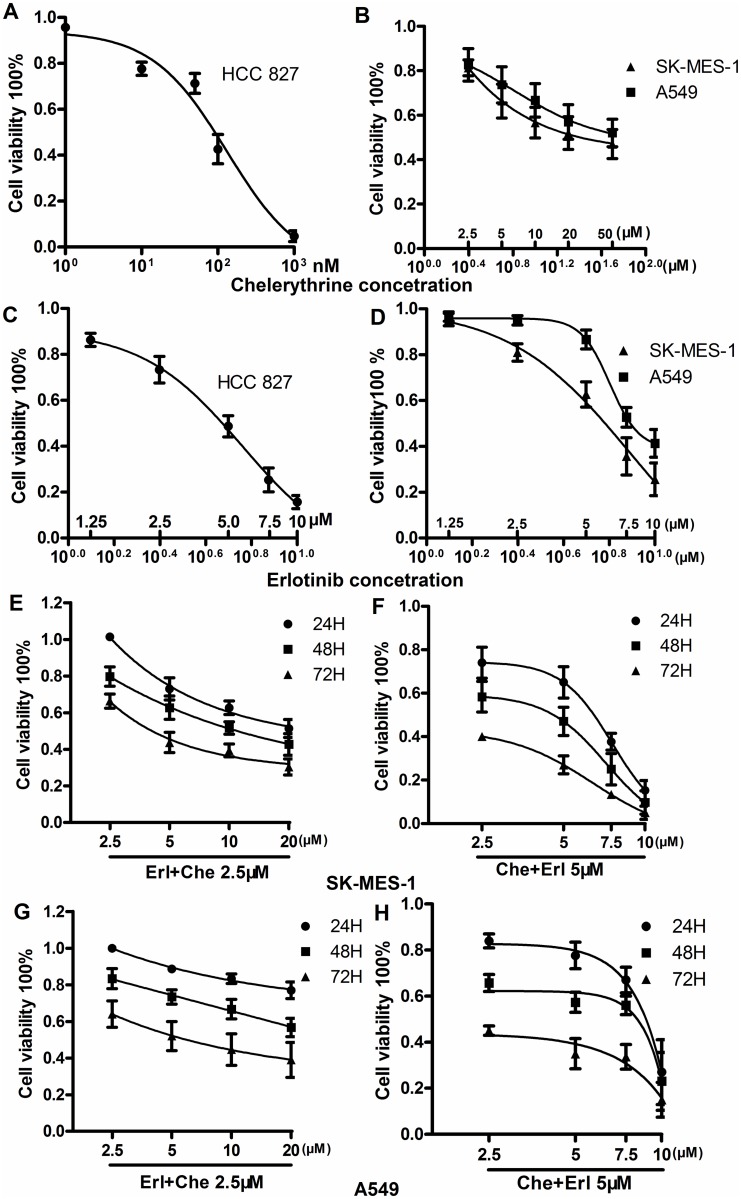
Effects of erlotinib (Erl) or/and cherlerythrine (Che) on the viability of NSCLC cells. A to D: IC_50_ of both compounds on HCC827, SK-MES-1 and A549 cells was assessed by alamar blue assay at 48 hours after drug treatment as described in the methods section. After IC_50_ of each compound was identified, the combination effect on cell viability was assessed on erlotinib less sensitive SK-MES-1 and A549 cells at 24, 48 and 72 hours after treatment. E and F: The combination effect on SK-MES-1 cell growth. G and H: The combination effect on A549 cell growth. The fluorescence value was recorded at a range from 540nm to 590nm. The percentage of cell growth was calculated as following: cell growth (%) = (experiment well/control well) x 100%; n = 3. Mean ± SD. N = 3.

The effects of erlotinib or/and chelerythrine on NSCLC cell lines were assessed using alamar blue assay. Compared to HCC827 cells (IC_50_ = 131±1.01nM), both SK-MES-1 and A549 cells showed a significantly less sensitivity to erlotinib, where the IC_50_ for SK-MES-1 was 43.42±4.35μM and for A459 was 49.88±0.47μM. (Fig 1A & 1B) (p<0.001 for all). As a comparison, there appeared to be no significant changes of IC_50_ for chelerythrine between the three cell lines. The IC_50_ of chelerythrine for HCC827, SK-MES-1 and A459 was 5.0±0.48μM, 6.35±1.26μM and 7.78±0.56μM, respectively (Fig 1C & 1D). When compared with erlotinib, chelerythrine showed potentiated inhibitory effects, particularly on erlotinib less sensitive SK-MES-1 (Fig 1E & 1F) and A459 cells (Fig 1G & 1H). S2 Fig illustrates the structure of chelerythrine.

### Combination of chelerythrine and erlotinib reduced NSLCC cell viability and colony formation

To elucidate the cytotoxicity induced by chelerythrine, whether chelerythrine has additive effects to erlotinib less sensitive SK-MES-1 and A549 cells was next evaluated. The cell viability in different combination modules was measured: 1) various doses of erlotinib and a constant dose of chelerythrine; and 2) various doses of chelerythrine with a constant dose of erlotinib. Compared with either the erlotinib or chelerythrine group treated alone, the combination of erlotinib and chelerythrine significantly reduced cells viability in a time- and dose-dependent manner for both SK-MES-1 (Fig 1C & 1D) and A549 cells (Fig 1E & 1F). The CI of SK-MES-1 and A549 was 0.98 and 1.08, respectively. The Bliss independence criterion analysis also confirmed an additive effect of chelerythrine to erlotinib less sensitive cells. Based on the effectiveness on cell viability, the concentrations used in subsequent experiments were 5μM of erlotinib combined with 5μM of chelerythrine on SK-MES-1 cells or 5μM of erlotinib combined with 7.5μM of chelerythrine on A549 cells. In addition, cell viability of HCC827 was significantly reduced with the combination of erlotinib (10nM) and chelerythrine (2.5μM) compared with the control or single compound groups (S1 Fig).

**Fig 2:**
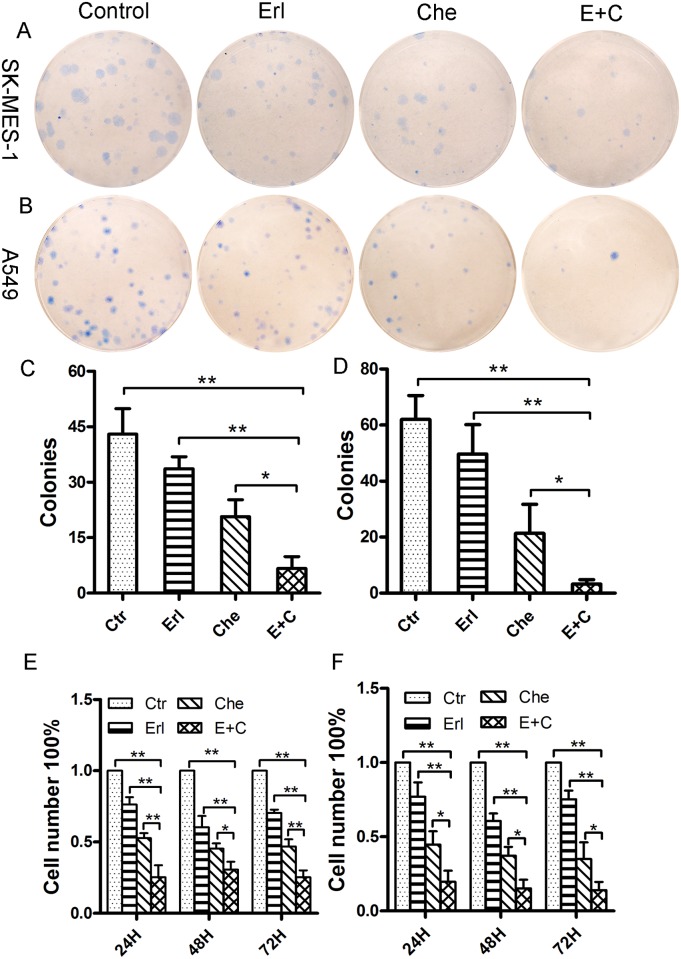
The combination of chelerythrine and erlotinib significantly inhibited cell colony formation and proliferation in SK-MES-1 and A459 cells. A and B: Cells were treated either with erlotinib (5μM), chelerythrine (5μM for SK-MES-1, and 7.5μM for A549) or the combination (E+C) of both for 24 hours. Fresh growth medium replaced the medium with the drugs and the cells were incubated for 12 days. After that, cells were fixed with methanol and stained with methylene blue. C and D: The colonies including >50 cells were calculated (C: SK-MES-1, D: A549). E and F: Cell number count for proliferation. The same number (0.4×10^5^) of SK-MES-1 (Fig. 2E) and A549 (Fig. 2F) cells were cultured for 24 hours. The medium was replaced with fresh medium containing erlotinib (5μM), chelerythrine (5μM for SK-MES-1, and 7.5μM for A549) or a combination of both. Direct cell number counting was performed after 24, 48, and 72 hours. N = 3, Mean ± SD, *: P<0.05, **: P<0.01.

The cytotoxicity effects of the combination of chelerythrine with erlotinib were further accessed by cell colony formation assay (Fig 2A & 2B) and directly by cell counting. Compared with the control group and the erlotinib or the chelerythrine treated alone, cell colonies were significantly reduced in the combination treated groups, resulting in a 35–55% reduction across all two NSCLC lines (Fig 2C & 2D) (p = 0.041 & p = 0.033). The combination treatment also resulted in a significant number of cell reduction through all three time periods from 24 to 72 hours for both cell lines (Fig 2E for SK-MES-1 and Fig 2F for A549) (p = 0.004 & p = 0.035).

### Combination of chelerythrine and erlotinib reduced NSLCC cell migration and invasion

**Fig 3:**
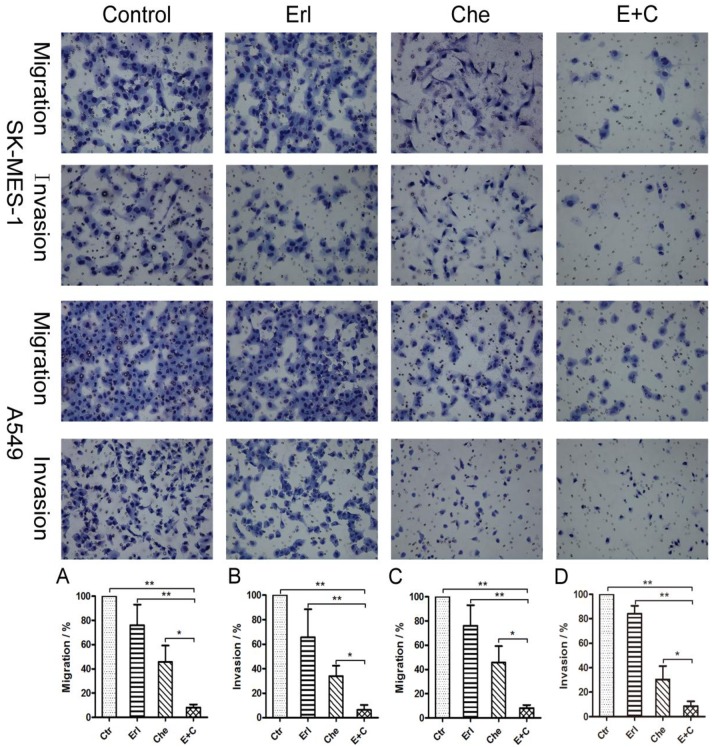
The combination treatment of chelerythrine and erlotinib significantly reduced cell migration and invasion in SK-MES-1(Fig. 3A & B) and A459 (Fig. 3C & D) cells. Viable cells were collected and counted after being treated with erlotinib, chelerythrine or a combination for 24h. The 4×10^5^ cells were plated into each insert and cultured for 20 hours. Cells were fixed and stained immediately. A and B: Migration and invasion of SK-MES-1 cells. D and E: Migration and invasion of A549 cells. N = 3, Mean ± SD, *: P<0.05, **: P<0.01.

To determine the combination affect of cell migration or invasion, *in vitro* trans-well migration and invasive assays were performed. The relative migration and invasion rate of SK-MES-1 (Fig 3A & 3B) were 11.67% and 6.33%, respectively. For the A549 cells (Fig 3C & 3D) the relative migration and invasion rates were 8.00% and 8.67%, respectively. Compared to the control or single drug treatment, the combination significantly reduced migration and invasion of both SK-MES-1 and A549 cells.

### Effects of erlotinib or/and chelerythrine on cell apoptosis

**Fig 4:**
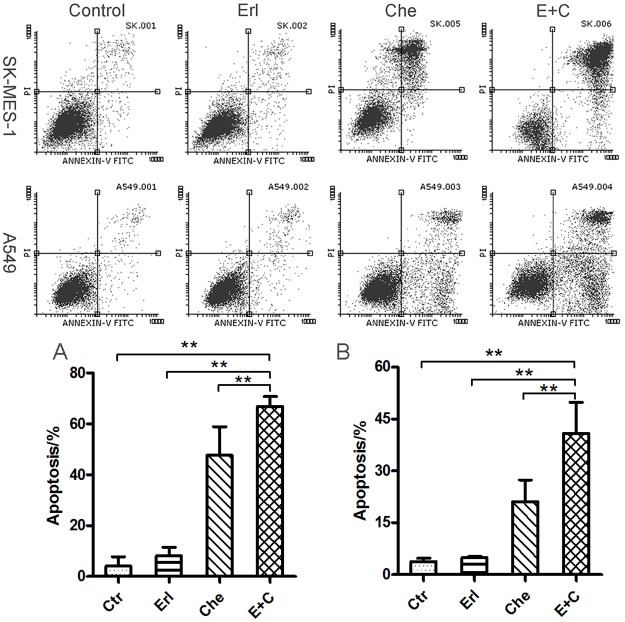
The combination of chelerythrine and erlotinib significantly induced apoptosis in SK-MES-1 (Fig. 4A) and A549 (Fig. 4B) cells. Cells were harvested after treatment for 24 hours, washed twice with cold PBS, and re-suspended in a 500μl binding buffer for a final cell concentration of 1-5x10^5^cells/ml. They were incubated in the dark with 5μl of FITH Annexin V and 5 μlPI (20 μg/ml) for 30min. Subsequently, the suspension was analyzed by FCM. N = 3, Mean ± SD, **: P<0.01.

To understand the mechanisms of erlotinib or/and chelerythrine on cell growth inhibition, FCM was used to analyze apoptosis. After treatment for 24 hours, the apoptosis rates of the control SK-MES-1 cells, erlotinib, chelerythrine and combination group were 4.12%, 8.11%, 47.63% and 66.90% (Fig 4A), respectively. The apoptosis rates of A549 in each group were 3.82%, 4.97%, 21.05% and 40.83% (Fig 4B), respectively. The apoptosis rate in the combination treatment group increased significantly in comparison to the control group and single drug treatment groups (P<0.05, n = 3). Additionally, cell cycle analysis was performed under the same conditions, and it was found that there were no significant difference in any phases in any of the groups (data not shown).

### Combination of chelerythrine and erlotinib inhibited EGFR signaling and phosphorylation of downstream targets in vitro

**Fig 5:**
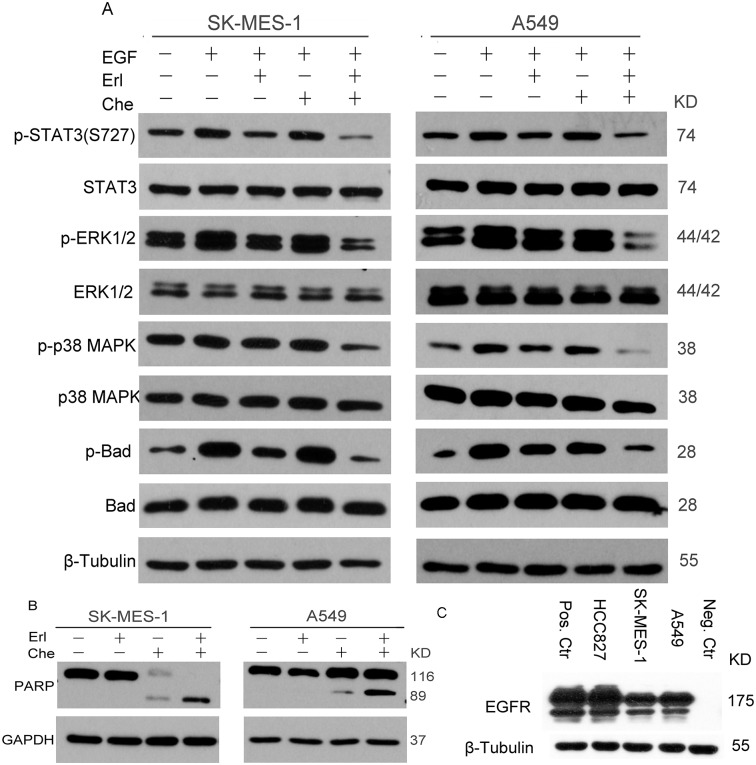
The combination of chelerythrine and erlotinib significantly inhibited EFGR downstream signal and activated PARP. A. Cells were treated with eroltinib, chelerythrine or combination of both in a serum free medium for 12 hours before stimulation with EGF for 10 minutes. Cell lysates were collected for western blot analysis of p-STAT3 (S727)/STAT3, p-ERK1/2/ERK1/2, p-p38 MAPK/p38 MAPK, p-Bad/Bad, and β-tubulin was used as a loading control. B. Activation of the poly (ADP-ribose) polymerase (PARP). Cells were treated with eroltinib, chelerythrine or a combination of both in the growth media for 24 hours. Cell lysates were collected for western blot analysis of PARP, and GAPDH was used as a loading control. 3 independent experiments were performed. C. EGFR expression on three NSCLC cells.

To further elucidate the mechanism of erlotinib or/and chelerythrine on the EGFR signal pathway in SK-MES-1 and A549 cell lines, each group (control, erlotinib, chelerythrine and combination) was treated in a serum free medium for 12 hours before stimulating with human EGF for 10 minutes, and then harvested for western blot analysis. The combination of chelerythrine and erlotinib inhibited EGF induced phosphorylation of STAT3, ERK1/2, p38 MAPK and Bad expressions, as shown in Fig 5A. Furthermore, the expression of PARP was upregulated by a combination of chelerythrine and erlotinib when cells treated in the growth media for 24 hours (Fig 5B). All three NSCLC cell lines expressed basal EGFR, but a higher level was detected in HCC827 cells (Fig 5C).

### Antitumor effects of erlotinib and/or chelerythrine in vivo

**Fig 6:**
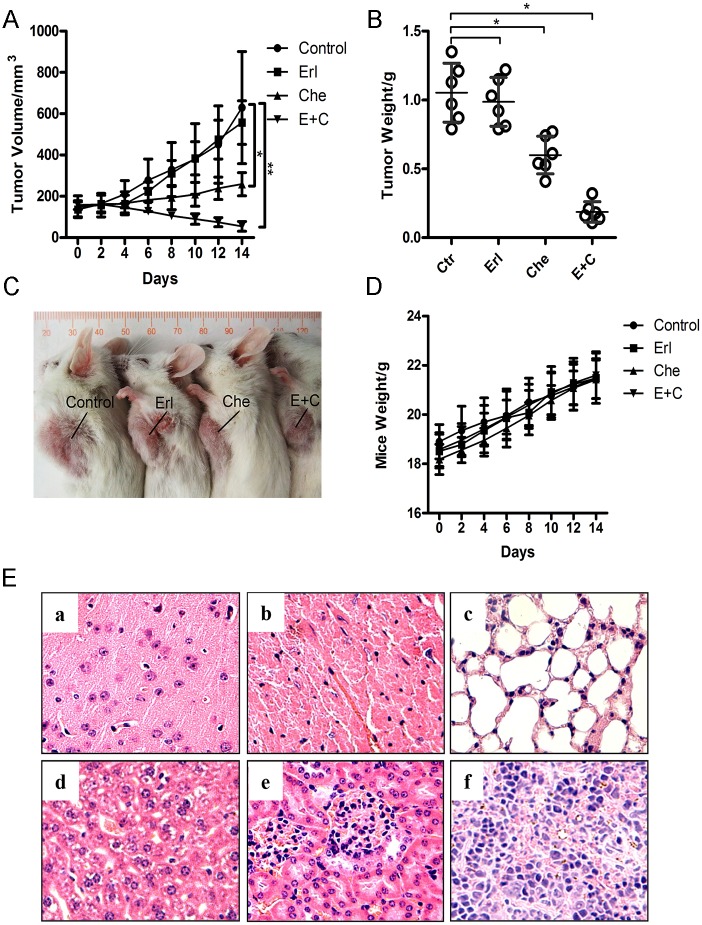
Effects of chelerythrine and erlotinib on SK-MES-1 xenograft mice model. A, B and C. NOD SCID mice were injected with 4×10^6^cells suspended in 0.1ml of PBS subcutaneously into the left chest. Treatments (erlotinib: 50mg/kg, o. p. daily; cheleryhine; 10mg/kg, o. p. daily; or a combination of both drugs; and DMSO was used as the control group) with drugs were initiated when the tumor volume reached about 150mm^3^. Tumor volume and mice weight were recorded every two days. At the end of the 14 day treatment, the mice were sacrificed, and the xenograft tumors were taken out and weighed. N = 6; Mean ± SEM; (E+C vs Control was considered very significant at Day14, **p = 0.0012; Che vs Control was also significant, *p<0.05; Two-way ANOVA). D. mice weights were monitored during the treatment. E. normal histologically appearance on the organs from a representative mouse treated with a combination of chelerythrine and erlotinib.

The effects of erlotinib, chelerythrine, and chelerythrine combined with erlotinib treatment on tumor growth was evaluated. SK-MES-1 cells were injected subcutaneously into NOD SCID mice. As shown in Fig 6, erlotinib treatment alone delayed tumor growth during the first 8 days, then quickly approached to the same volume as with the control group, while the chelerythrine treatment alone significantly inhibited tumor growth by an average volume of 210mm^3^ v.s. 610mm^3^ in the control at day 14 post-treatment (p = 0.024). The chelerythrine combined with the erlotinib group however, had significant tumor growth delay, whereby they induced a significant reduction in tumor volume (Fig 6A–6C), the effect of E+C vs Control was considered very significant at Day14, **p = 0.0012; E+C vs Erl was considered significant at Day14, *p<0.05. Che vs Control was also significant, p<0.05. Tumor weight was also deducted (Fig 6B; Che v.s. Ctr, and E+C v.s. Ctr, *p<0.05). All three treatment regimens were well tolerated, and the mice body weights remained unchanged over the duration of these experiments. The mice organs from each treatment regimens were essential normal histologically (Fig 6D & 6E).

## Discussion

To investigate the effects of the combination of chelerythrine with erlotinib on NSCLC cell lines (SK-MES-1 and A549), cell viability, proliferation, migration, invasion and apoptosis assays were performed *in vitro* and tumor growth experiments *in vivo*. The results showed that there was an additive effect of chelerythrine to erlotinib less sensitive SK-MES-1 and A549 cell lines. The combination treatment of chelerythrine and erlotinib significantly decreased cell proliferation, reduced clonogenicity, migratory and invasive capabilities and induced apoptosis, along with a significant delay of tumor growth. The possible mechanism was the combination of the treatment being highly effective at blocking EGF induced phosphorylation of STAT3, ERK1/2, p38 MAPK and Bad proteins.

Erlotinib as an EGFR tyrosine kinase inhibitor (TKI) has been widely used in clinical cases. In lung cancer, it has been shown to be more effective in patients with EGFR mutation. When compared with an EGFR mutated cell line HCC827, both SK-MES-1 and A459 cells showed lower levels of EGFR expression (Fig 5C) and were less sensitive to erlotinib (Fig 1A & 1B). Furthermore, when the cells treated with both erlotinib and chelerythrine, an additive antitumor effect was observed in cell proliferation (Fig 1E–1G), clonogenicity (Fig 2C & 2D), and migration and invasion (Fig 3A–3D) in both SK-MES-1 and A459 cells. In vivo experiments further confirmed that the additive effect delayed tumor growth in the combined treatment group (Fig 6A–6C).

The major pathways activated by EGF in NSCLC include Ras/Raf/MAPK, PI3K/AKT, PLCγ and STATs signaling. All of which lead to the transcription of target genes that may contribute to NSCLC progression. Those pathways have been studied extensively in NSCLC signaling [18,19]. Several reports showed that the inhibition of STAT3 suppressed the growth of cancer cells and enhanced the sensitivity to anticancer agents in multiple types of cancer [20–22]. Overexpression of total or phosphorylated STAT3 in resected NSCLC leads to a poor prognosis. In a preclinical study, the overexpression of STAT3 was correlated with chemoresistance and radioresistance in NSCLC cells [23,24]. Additionally, PKC activates Raf, which leads to MAPK activation [25]. Indicated in the presented results the main downstream mediators of the EGFR signal transduction pathway were affected by the chelerythrine and erlotinib combination, which significantly reduced both STAT3 and MAPK phosphorylation (Fig 5A). Moreover, MAPK downregulation correlated with the enhancement of PI3K/AKT signaling since the phosphor of the drug induced apoptosis and antitumor activity in lung cancer cells. The reduction of activated MAPK may explain the increased apoptosis found in the chelerythrine and erlotinib combination (Fig 4A & 4B). However, the experiment was not able to detect the changes of phosphorylation in AKT and JNK (data not shown).

The presented data showed the combination reduced the p-Bad expression significantly. Bad is one of the ‘death-promoting’ members of the Bcl-2 family, which only has a BH3 domain, and its pro-apoptotic activity is regulated primarily by phosphorylation at several sites [26]. Activated ERK1/2-p90 ribosomal S6 kinase-1 (p90Rsk-1) [27,28] and AKT [29,30] pathways have been shown to promote survival signaling by phosphorylating Bad at Ser136 and Ser112, respectively. Previous studies indicated that either TKI or PKC inhibitors showed the inhibition in Bad expression. Gilmore reported that the phosphorylation of Bad-Ser112 via the ERK1/2 pathway is inhibited by either gefitinib or the MEK inhibitor PD98059 in mammary epithelial cells and primary cultures of malignant breast carcinoma [31]. Axelrod treated KU-7 cells with the EGFR inhibitor lapatinib and p70S6K inhibitor AT7867 simultaneously, has found that the combination leads to apoptosis that may be mediated through reducing Bad phosphorylation [32]. Chan reported the identification of chelerythrine as an inhibitor of BclXL-Bak BH3 peptide binding [33]. Furthermore, BclXL-overexpressing cells that were completely resistant to apoptotic stimuli used in the study remained sensitive to chelerythrine. Another study reported theinduction of apoptosis by chelerythrine through mitochondrial pathways and the Bcl-2 family proteins in the human hepatoma SMMC-7721 cell [34].

Here data depicted that inhibiting both EGFR and PCK signaling could reduce migration and invasion in the NSCLC cell. Increasing evidence suggests that chemotaxis plays a pivotal role in the progress of tumor metastasis. Ying’s study suggested that PKCζ was required in NSCLC cell chemotaxis, and thus could be used as a target to develop anti-lung cancer metastasis therapies [35]. Iwabu demonstrated that EGF stimulation increased myosin light chain (MLC) phosphorylation, a marker for the contractile force, and a concomitant with PKC activity in mouse fibroblasts expressing human EGFR constructs [36].

The success of single-agent antitumor therapy can be limited by downstream mutations or the induction of parallel oncogenic pathways in tumor cells [37]. Resistance can be caused by the activity of alternative signaling pathways that compensate for the pathways being inhibited by the therapeutic agent [38]. The presented findings on the combination of chelerythrine and erlotinib agree with those reported by Abera et al., who found that the elevation of PKCα expression as well as PKCα-dependent downregulation of PKCδ are required for erlotinib resistance. It’s a potential therapeutic use of PKCα inhibitors to overcome drug resistance and epithelial-to-mesenchymal transition (EMT) in lung cancer [6].

Chelerythrine has specific inhibiting activities against PKC and has been reported in the induction apoptosis in many cell types. In osteosarcoma, chelerythrine induced apoptosis is mediated through the activation of the RAF/MEK/ERK pathway [39]. In the presented study, significant inductions of apoptosis have been observed in the combination treatment by chelerythrine with erlotinib in both erlotinib-less sensitive cells (Fig 4A & 4B). However, the activation of the RAF/MEK/ERK pathway by chelerythrine was not observed when NSCLC cells were stimulated with EGF together (Fig 5A). A recent study conducted by Yang *et al*. emphasized the poor selectivity of chelerythrine comparing tumor and non-tumor cell lines [35]. Yamamoto’s study suggested that intravenous injection of chelerythrine activates caspases and promotes apoptosis in adult rat hearts *in vivo* [40]. While Medvetz [41] reported mice bearing TSC2-null xenograft tumors were treated daily with chelerythrine (10mg/kg/day) for 4 weeks and no effect on body weight or other evidence of toxicity was observed. Here it is shown that all three treatment regimens were well tolerated; the mouse organs from each treatment groups were essential normal histologically when gavaging either chelerythrine (10mg/kg/day) alone or when combined with erlotinib for 14 days. The current study is still limited to exploring how chelerythrine prevents or delays the acquisition of the resistance in cell lines sensitive to erlotinib or in exploring which specific or some of the subtypes of PKC play critical role in the erlotinib less sensitive cell. These will be directions for future study.

In conclusion, the presented study aids in the understanding of the combination effect of chelerythrine with erlotinib in NSCLC, and provides new insight into antitumor combinations for lung cancer therapy references. These findings are important since chelerythrine might be a promising agent to improve the effect of erlotinib in patients with erlotinib less sensitive lung cancer. This is the first report known suggesting the additive effects of the PKC inhibitor chelerythrine in combination with EGFR-TKI erlotinib on human NSCLC.

## Supporting information

S1 Fig Cell viability analysis for HCC827 cells. (TIF)

S2 Fig The structures for chelerythrine chloride (A) and erlotinib (B). (TIF)

## Abbreviations

aPKC: atypical protein kinase C
CI: combination index
EGFR: epidermal growth factor receptor
MAPK: mitogen-activated protein kinase
MLC: myosin light chain
NSCLC: non-small-cell lung cancer
PI3K: phosphatidylinositol 3-kinase
PKC: protein kinase C
SQCLC: squamous-cell carcinoma
STAT3: signal transducer and activator of transcription 3
TKI: tyrosine kinase inhibitor
TNFα: tumor necrosis factor–α
