# Supplementary figures and images for: Additive effects of cherlerythrine chloride combination with erlotinib in human non-small cell lung cancer cells
Source: PLoS One. 2017 Apr 11;12(4):e0175466. doi: 10.1371/journal.pone.0175466 (PMC5388488; doi:10.1371/journal.pone.0175466)

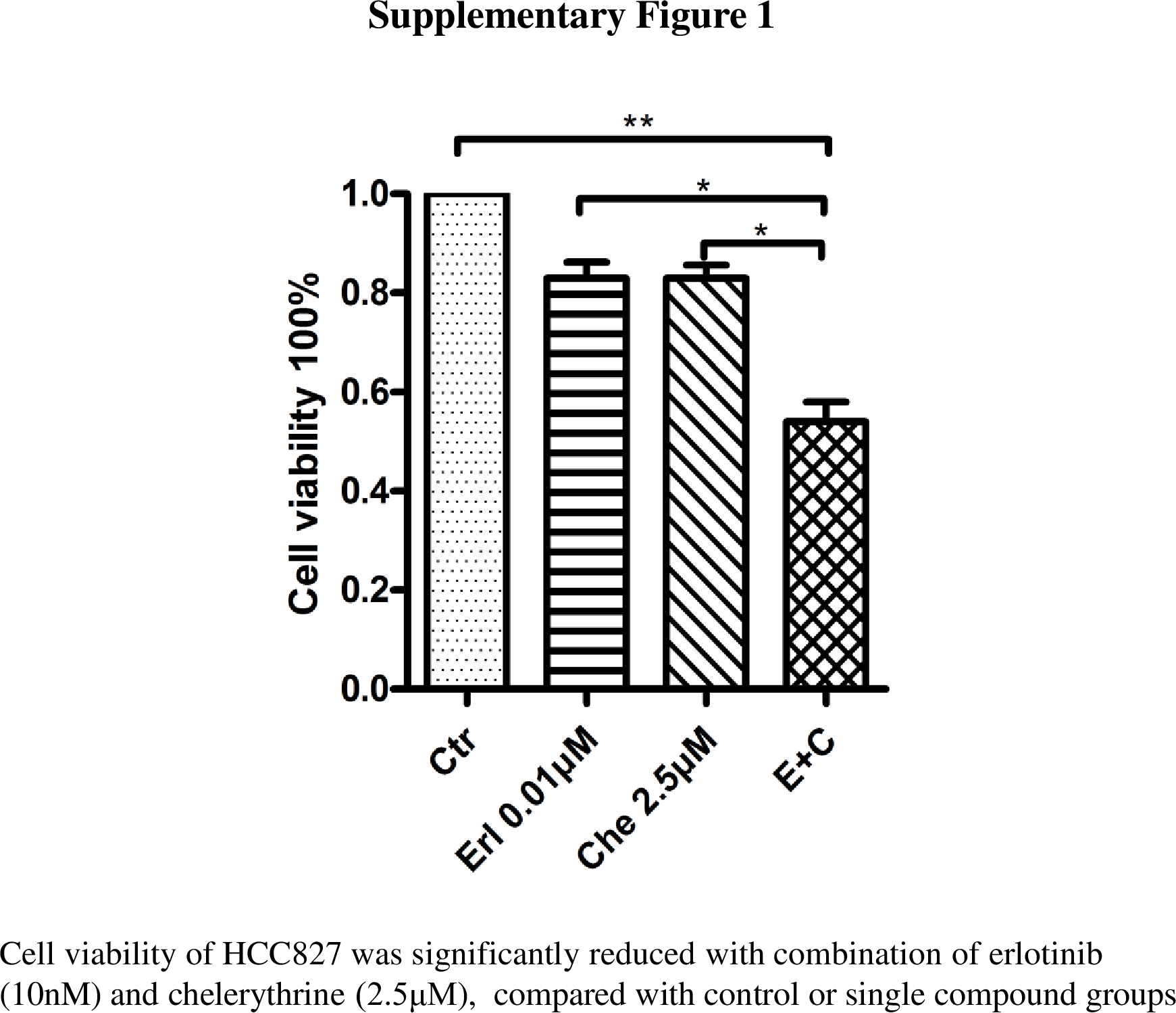

Supplement: S1 Fig — (TIF) [file pone.0175466.s001.tif]

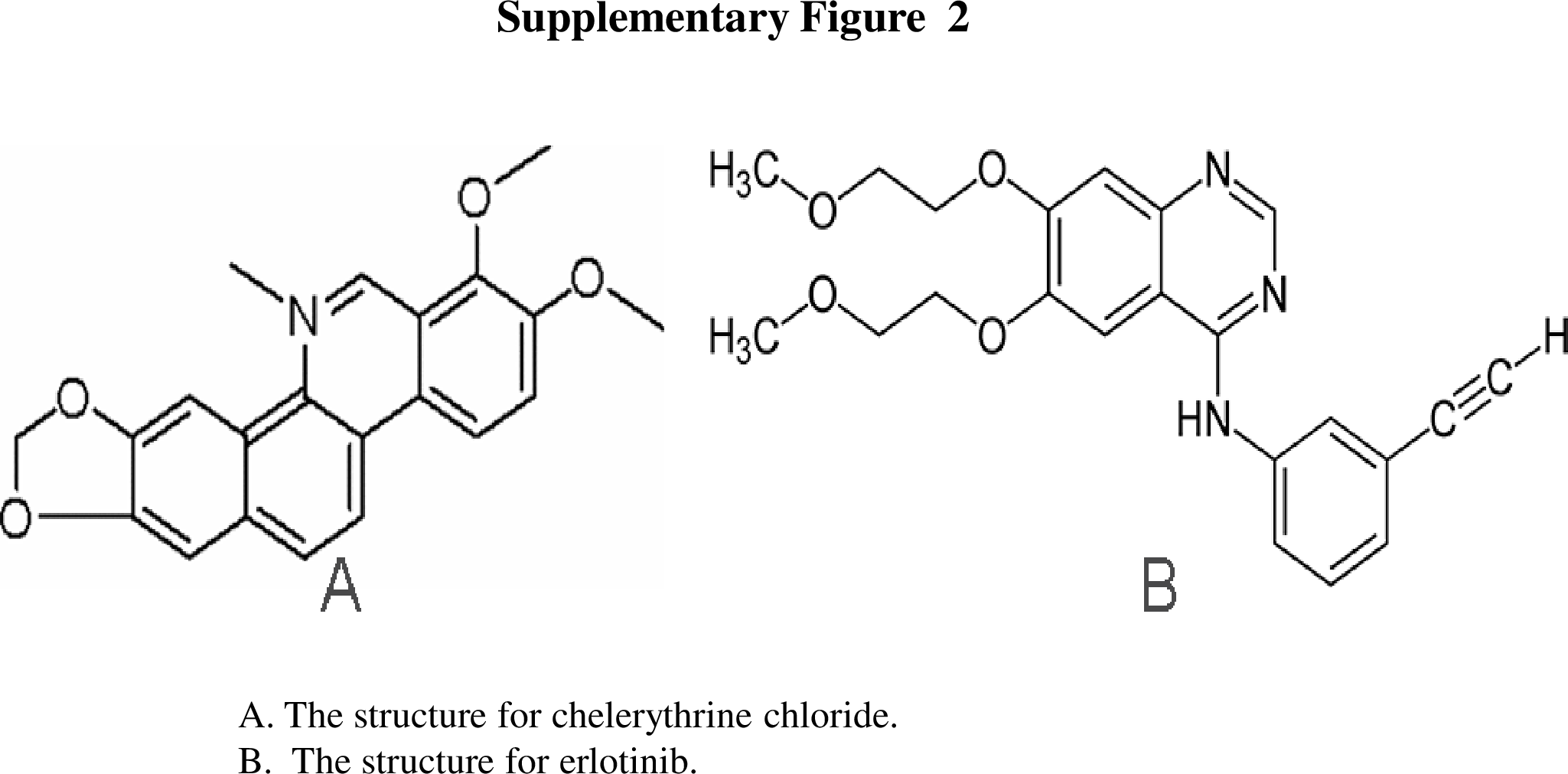

Supplement: S2 Fig — (TIF) [file pone.0175466.s002.tif]
